# Early arrhythmia recurrence after catheter ablation for persistent atrial fibrillation: is it predictive for late recurrence?

**DOI:** 10.1007/s00392-021-01934-8

**Published:** 2021-09-14

**Authors:** Miruna A. Popa, Marc Kottmaier, Elena Risse, Marta Telishevska, Sarah Lengauer, Katharina Wimbauer, Amir Brkic, Verena Kantenwein, Stephanie Ulrich, Marielouise Kornmayer, Hannah Krafft, Monika Hofmann, Susanne Kathan, Tilko Reents, Isabel Deisenhofer, Gabriele Hessling, Felix Bourier

**Affiliations:** Department of Electrophysiology, German Heart Center Munich, Technical University Munich, Lazarettstr. 36, 80636 Munich, Germany

**Keywords:** Persistent atrial fibrillation, Catheter ablation, Early recurrence, Blanking period

## Abstract

**Background:**

Early recurrence of atrial tachyarrhythmia (ERAT) is common after radiofrequency catheter ablation (RFCA) for atrial fibrillation (AF), but its clinical significance in patients with persistent AF remains unclear. We sought to determine the predictive value of ERAT for rhythm outcome after RFCA for persistent AF.

**Methods:**

The study included 207 consecutive patients (mean age 66.4 ± 10.7 years, male 66.2%) with persistent and long-standing persistent AF undergoing de novo pulmonary vein isolation (± atrial substrate ablation). All patients remained off antiarrhythmic drugs. ERAT was defined as any atrial arrhythmia ≥ 30 s occurring within the first 30 days. Late recurrence (LR) was determined during follow-up visits scheduled 1, 3, 6 and 12 months post-ablation using 7-day Holter ECGs.

**Results:**

ERAT occurred in 143/207 (69.1%) patients as AF (60%) or atrial tachycardia (40%) and was persistent in 82% of cases. During a median follow-up of 22.2 months, LR occurred significantly more often in patients with ERAT than in patients without ERAT (92.3 vs. 43.8%, *P* < 0.001). The only independent predictors for LR were ERAT (OR 16.8, 95% CI 6.184–45.797, *P* < 0.001) and intraprocedural termination to sinus rhythm (OR 0.052, 95% CI 0.003–0.851, *P* = 0.038). Extending the blanking period from 30 to 90 days did not impact LR rates.

**Conclusion:**

ERAT following ablation of persistent AF is strongly associated with late arrhythmia recurrence, which challenges the assumption that ERAT represents merely a transient phenomenon. While limiting the blanking period to 30 days seems justified, the benefit of early re-ablations remains to be addressed in future studies.

**Graphic abstract:**

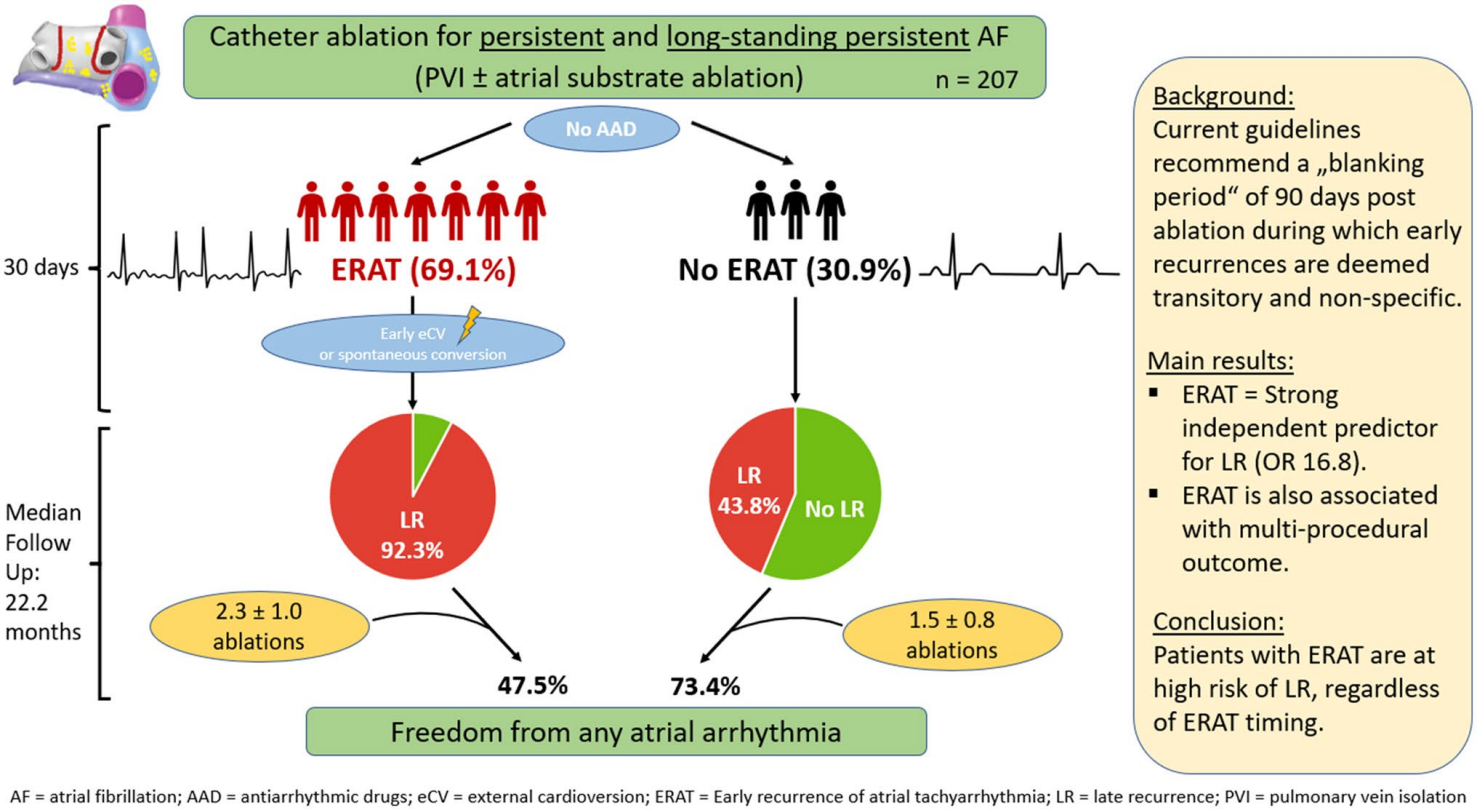

## Introduction

Radiofrequency catheter ablation (RFCA) has become an established therapy option for patients with symptomatic drug-refractory atrial fibrillation (AF). Early recurrence of atrial tachyarrhythmia (ERAT) after pulmonary vein isolation (PVI) is common with a reported incidence of 40–60% [1–3]. This phenomenon is believed to be of transient nature due to reversible processes including post-ablation inflammation, short-term autonomic imbalance and lesion maturation time [4–6]. Therefore, a “blanking period” of 3 months following AF catheter ablation is generally recommended during which early recurrences are deemed non-specific and not regarded as treatment failure [7].

However, there is increasing evidence that ERAT following CA for paroxysmal AF is strongly associated with late recurrence (LR) beyond the blanking period [1, 2, 8]. In contrast, the prognostic significance of ERAT in patients with persistent and long-standing persistent AF is poorly defined, since the majority of data (~ 90%) originates from paroxysmal AF patient cohorts [9]. RFCA for persistent AF is associated with less favorable outcomes [7] and with higher ERAT rates [10–12] than for paroxysmal AF. Therefore, identifying patients with persistent AF at higher risk of developing arrhythmia relapse may help establish the optimal timing for re-ablation and improve clinical outcomes.

The current study sought to characterize ERAT occurring in the first month after RFCA for persistent AF and to determine its prognostic significance for long-term single- and multi-procedural outcomes.

## Methods

### Study population

Data of 207 consecutive patients (age ≥ 18 years) receiving de novo RFCA for drug-refractory symptomatic persistent or long-standing persistent AF at our institution from January 2015 to July 2016 was assessed. Baseline characteristics, procedural and follow-up data were collected prospectively and recorded in a computerized database. All patients showed AF duration ≥ 4 weeks, presented in AF at the time of ablation and had discontinued antiarrhythmic drugs (AADs) > 5 half-lives before the procedure. Patients presenting in sinus rhythm (SR), with a history of heart surgery requiring atriotomy or with prior left atrial (LA) ablation were not included in the study. Persistent and long-standing persistent AF were defined according to current guidelines [7]. AF history was defined as the time span from the first diagnosis of AF. AF duration was defined as the longest AF episode. All patients provided written informed consent for the procedure and the study was approved by the local ethics committee (approval # 348/20 S-SR).

### Catheter ablation procedure

All patients received uninterrupted oral anticoagulation (direct oral anticoagulants or phenprocoumon with an international normalized ratio between 2 and 3) for at least 4 weeks prior to the procedure. LA thrombus was excluded by either contrast-enhanced computed tomography or transesophageal echocardiogram < 48 h prior to ablation. Electrophysiological study was performed under conscious sedation with propofol, fentanyl and midazolam. Heparin was administered as continuous infusion following transseptal puncture to gain an intraprocedural activated clotting time > 300 s.

Vascular access was obtained using the right femoral vein. Double access to the LA was gained by fluoroscopy-guided single transseptal puncture with a steerable 11.7-Fr sheet (Agilis^™^, St. Jude Medical, Minneapolis, MN). Electro-anatomical mapping of the LA was performed using a circular mapping catheter (Orbiter PV variable loop, Boston Scientific, Marlborough, MA or Lasso Nav Eco 2515, Biosense Webster, Diamond Barr, CA) and a 3D mapping system (Ensite Velocity^™^, St. Jude Medical or Carto3, Biosense Webster). Wide antral circumferential PVI and atrial substrate ablation were performed with an open-irrigated-tip RF catheter (FlexAbility^™^, St. Jude Medical or ThermoCool SmartTouch^®^ SurroundFlow, Biosense Webster) using point-by-point-lesions (30–40 W for 30 s), as described previously [13]. Complete PVI was confirmed by documented elimination of all pulmonary vein (PV) potentials by the circular mapping catheter placed in each PV ostium and reassessed in SR at least 30 min after PVI completion.

If AF persisted after PVI, additional substrate modification (ablation of low voltage areas and fractionation) was performed with the endpoint of AF cycle length prolongation or AF termination into SR or atrial tachycardia (AT) [13]. Stable ATs were mapped and ablated; in case of macro-reentries, linear lesions were applied and bidirectional line block was subsequently tested. If neither termination to AT nor SR was achieved, electrical cardioversion was performed. Mean AF cycle length was measured in the LA appendage prior to PVI and after additional substrate ablation.

### Follow-up

All patients remained off AADs post-ablation. Patients received continuous in-hospital ECG monitoring during the first 48 h following CA. Follow-up was scheduled 1, 3, 6, and 12 months after the index procedure in our outpatient clinic and comprised clinical assessment, resting ECG and 7-day Holter ECG. Patients were seen in interim visits in case of symptoms suggestive of arrhythmia recurrence.

ERAT was defined as any documented atrial arrhythmia ≥ 30 s occurring during the first 30 days after RFCA. ERAT type and timing were assessed based on in-hospital ECG recordings, external resting and Holter ECGs, implanted device recordings (if available), and patient symptoms. In patients with persistent ERAT, electrical cardioversion was performed during the blanking period to promote reverse remodeling.

LR was defined as any atrial arrhythmia ≥ 30 s documented > 30 days post-ablation. Re-ablations were performed at least 3 months after the index ablation in case of documented symptomatic LR. In exceptional cases of highly symptomatic tachyarrhythmia recurrence with ≥ 1 unsuccessful electrical cardioversion, re-ablation was performed starting 6 weeks post-ablation.

### Statistical analysis

Continuous variables are expressed as mean ± standard deviation or median and interquartile range (25th and 75th percentile; in case of non-normal distribution) and compared by *t* tests. Categorical variables are presented as frequencies or percentages and compared by *χ*^2^ tests. Time to first LR was plotted using the Kaplan–Meier product limit method and compared by the log-rank test. Univariate und multivariate backward logistic regression analyses were performed to identify factors associated with ER and LR. Factors with a *P* value < 0.1 on univariate analysis were considered in the multivariate model. Cox regression analysis was performed to analyze the correlation between timing of first ERAT and risk of LR. Statistical tests and confidence intervals with 2-sided *P* < 0.05 were considered statistically significant. Statistical analysis was performed using the SPSS version 25.0 (IBM Inc., Armonk, NY).

## Results

### Baseline characteristics and factors associated with ERAT

A total of 207 patients with persistent and long-standing persistent AF (mean age 66.4 ± 10.7 years, male 66.2%) were followed for a median of 22.2 months (IQR 11; 41 months) after de novo RFCA. Baseline characteristics and procedural data are presented in Table 1. Median AF history and AF duration were 17 months (IQR 6; 47) and 6 months (IQR 4; 12), respectively. Long-standing persistent AF was noted in 63 (30.4%) patients.

**Table 1:** Baseline characteristics and procedural data of patients with and without early recurrence (ERAT)

	Univariate analysis for ERAT					
	No ERAT *n* = 64	ERAT *n* = 143	Total *n* = 207	Odds ratio	95% Confidence interval	*P* value
Clinical data						
Age, years	64.4 ± 11	67.3 ± 10	66.4 ± 10.7	1.025	0.998–1.054	0.073
Male, *n* (%)	50 (78.0%)	87 (60.8%)	137 (66.2%)	0.435	0.220–0.860	0.017
BMI, kg/m^2^	28.3 ± 4	28.5 ± 5	28.4 ± 4.5	1.008	0.944–1.076	0.815
Hypertension, *n* (%)	42 (65.6%)	104 (72.7%)	146 (70.5%)	1.397	0.741–2.632	0.301
Diabetes mellitus, *n* (%)	7 (10.9%)	23 (16.1%)	30 (14.5%)	1.561	0.633–3.850	0.334
Coronary artery disease, *n* (%)	22 (34.3%)	47 (32.8%)	69 (33.5%)	0.944	0.507–1.761	0.857
Previous stroke or TIA, *n* (%)	1 (1.6%)	9 (6.3%)	10 (4.8%)	4.231	0.525–34.127	0.176
Implanted device, *n* (%)	3 (4.7%)	17 (11.9%)	20 (9.7%)	2.743	0.774–9.719	0.118
ß blockers, *n* (%)	47 (73.4%)	112 (78.3%)	159 (76.8%)	1.307	0.660–2.586	0.442
ACEi/ARB, *n* (%)	40 (62.5%)	79 (55.2%)	119 (54.1%)	0.741	0.405–1.355	0.330
CHA_2_DS_2_VASc Score	2.1 ± 1.5	2.7 ± 1.5	2.5 ± 1.5	1.289	1.050–1.583	0.015
AF history, months*	13 (5.3;32.5)	18 (6;48)	17 (6;47)	1.002	0.995–1.009	0.527
AF duration, months*	5 (3;11.5)	7 (4;13)	6 (4;12)	1.015	0.993–1.037	0.186
LV-EF, %	56.2 ± 8	55.2 ± 7	55.5 ± 7.6	0.981	0.940–1.023	0.374
LA area, cm^2^	27.3 ± 6	29.3 ± 6	28.7 ± 6.1	1.059	1.005–1.116	0.032
Mitral valve insufficiency, moderate or severe, *n* (%)	6 (9.3%)	20 (14%)	26 (12.6%)	1.572	0.599–4.123	0.358
Creatinine, mg/dl	1.0 ± 0.2	1.0 ± 0.2	1.0 ± 0.2	0.636	0.171–2.371	0.500
Glomerular filtration rate, ml/min	76.8 ± 17.2	75.4 ± 18.2	75.8 ± 17.9	0.996	0.979–1.012	0.607
Procedural data						
Procedure time, min	182.2 ± 48.3	180.6 ± 44.1	181.1 ± 45.3	0.999	0.993–1.006	0.809
Radiofrequency time, min	66.0 ± 18.4	66.1 ± 18.8	66.1 ± 18.6	1.000	0.985–1.016	0.969
Pulmonary vein isolation, *n* (%)	64 (100%)	143 (100%)	207 (100%)	–	–	> 0.999
Substrate ablation, *n* (%)	62 (96.9%)	138 (96.5%)	200 (96.6%)	0.890	0.168–4.715	0.891
Linear lesions, *n* (%)	10 (15.6%)	16 (11.2%)	26 (12.6%)	0.680	0.290–1.595	0.375
CL difference between baseline and after substrate ablation, ms	37.4 ± 20	34.1 ± 29	35.1 ± 26.6	0.995	0.984–1.008	0.458
Termination to AT or SR, *n* (%)	16 (25%)	26 (18.1%)	42 (20.3%)	0.667	0.329–1.353	0.261
Termination to AT, *n* (%)	10 (15.6%)	18 (12.6%)	28 (13.5%)	0.778	0.337–1.795	0.556
Termination to SR, *n* (%)	12 (18.7%)	18 (12.6%)	30 (14.5%)	0.624	0.281–1.387	0.247
Follow-up data						
Late recurrence, *n* (%)	28 (43.8%)	132 (92.3%)	160 (77.3%)	–	–	< 0.001
Time to late recurrence, days	420.1 ± 333.7	155.5 ± 231.5	201.8 ± 270.6	–	–	< 0.001
Follow-up, months*	21.4 (12;41)	22.2 (9.3;42.3)	22.2 (10.7;41)	–	–	0.567

Multivariate analysis for ERAT			
Predictor	Odds ratio	95% Confidence interval	*P* value
Age	0.994	0.955–1.035	0.783
Male	0.539	0.255–1.140	0.106
CHA_2_DS_2_VASc Score	1.231	0.896–1.690	0.199
LA area	1.057	1.002–1.115	0.043

*ACEi* angiotensin-converting-enzyme inhibitors; *ARB* angiotensin II receptor type 1 blockers; *AT* atrial tachycardia; *BMI* body mass index; *CL* cycle length; *ERAT* early recurrence of atrial tachyarrhythmia; *LV-EF* left-ventricular ejection fraction; *SR* sinus rhythm; *TIA* transient ischemic attack

*Non-normally distributed continuous variables are expressed as median and interquartile range (25th and 75th percentile)

**Fig. 1:**
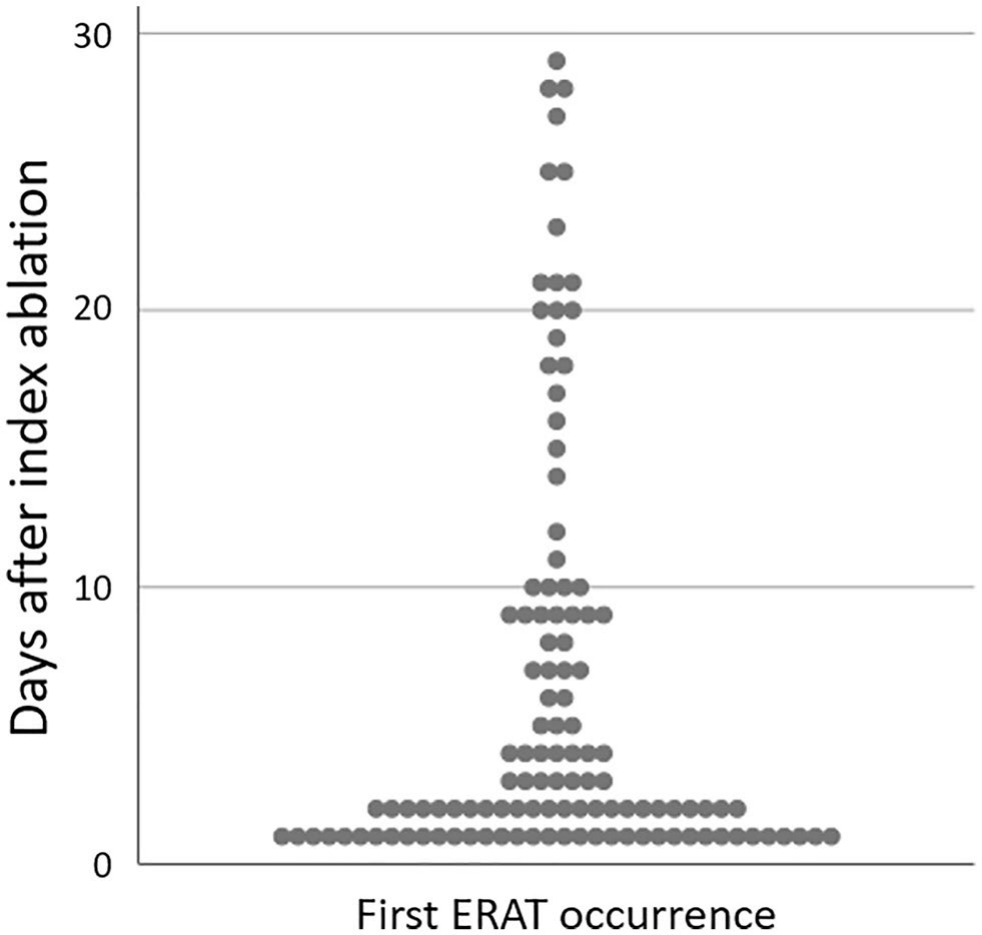
Timing of first ERAT occurrence presented as a scatterplot. Exact ERAT onset could be determined in 118/143 (82.5%) patients

ERAT occurred in 143/207 patients (69.1%) after a median of 2 days (IQR 1; 9 days) as either AF (86/143; 60%) or atrial tachycardia (AT; 57/143; 40%). ERAT was persistent in 117/143 (82%) patients, while 26/143 (18%) patients experienced spontaneous conversion to SR. Highest ERAT incidence was observed in the first week following ablation (Fig. 1). First ERAT episode occurred in 42% of patients within 48 h and in 58% of patients within 7 days.

Patients with ERAT were more commonly female (39.2% vs. 22%, *P* = 0.017), had larger LA area (29.3 ± 6 vs. 27.3 ± 6 cm^2^, *P* = 0.032) and a higher CHA_2_DS_2_VASc Score (2.7 ± 1.5 vs. 2.1 ± 1.5, *P* = 0.015) compared to patients without ERAT. Age, AF history/duration and procedural data were not significantly different between groups. Additional substrate ablation did not significantly impact ERAT rates (Table 1). The LR rate was significantly higher in patients with ERAT (92.3 vs. 43.8%, *P* < 0.001) than in patients without ERAT. On multivariate analysis, LA area was the only independent predictor for ERAT (OR 1.057, 95% CI 1.002–1.115, *P* = 0.043).

### Predictors of late recurrence (LR) after single ablation procedure

During the median follow-up of 22.2 months, LR of any atrial tachyarrhythmia after a single ablation procedure occurred in 160 patients (77.3%). Initial LR presented as AF in 100/160 (62.5%) patients and AT in 60/160 (37.5%) patients. On univariate analysis, patients with LR had a higher age (68.1 ± 9.7 vs. 60.8 ± 12.0 years, *P* < 0.001), were more commonly female (38.2 vs. 19.2%, *P* = 0.018), had a longer mean AF duration (14.3 ± 22.5 vs. 6.7 ± 6.6 months, *P* = 0.022), presented more frequently mitral valve insufficiency (15.6 vs. 2.1%, *P* = 0.038) and had larger LA area (29.3 ± 6.1 vs. 26.7 ± 5.8 cm^2^, *P* = 0.014), compared to patients without LR (Table 2).

**Table 2:** Baseline characteristics and procedural data of patients with and without late recurrence

Univariate analysis for late recurrence					
	No late recurrence *n* = 47	Late recurrence *n* = 160	Odds ratio	95% Confidence interval	*P* value
Clinical data					
Age, years	60.8 ± 12.0	68.1 ± 9.7	1.064	1.031–1.099	< 0.001
Male, *n* (%)	38 (80.8%)	99 (61.8%)	0.384	0.174–0.850	0.018
BMI, kg/m^2^	27.8 ± 3.4	28.6 ± 4.8	1.042	0.967–1.123	0.281
Hypertension, *n* (%)	28 (59.6%)	118 (73.7%)	1.906	0.965–3.766	0.063
Diabetes mellitus, *n* (%)	3 (6.4%)	27 (16.9%)	2.977	0.861–10.295	0.085
Coronary artery disease, *n* (%)	14 (29.8%)	55 (34.4%)	1.197	0.590–2.430	0.618
Previous stroke or TIA, *n* (%)	1 (2.1%)	9 (5.6%)	2.742	0.338–22.215	0.345
Implanted device, *n* (%)	1 (2.1%)	19 (11.9%)	6.199	0.807–47.587	0.079
ß blockers, *n* (%)	37 (78.7%)	122 (76.3%)	0.868	0.395–1.908	0.724
ACEi/ARB, *n* (%)	25 (53.2%)	94 (58.8%)	1.253	0.652–2.410	0.498
CHA_2_DS_2_VASc Score	1.8 ± 1.5	2.7 ± 1.4	1.584	1.242–2.020	< 0.001
AF history, months*	10 (4;25)	19 (7;49.5)	1.011	1.000–1.022	0.040
AF duration, months*	4 (3;10)	7 (4; 13)	1.063	1.009–1.120	0.022
LV-EF, %	56.9 ± 8.1	55.1 ± 7.4	0.961	0.911–1.015	0.153
LA area, cm^2^	26.7 ± 5.8	29.3 ± 6.1	1.077	1.015–1.142	0.014
Mitral valve insufficiency, moderate or severe, *n* (%)	1 (2.1%)	25 (15.6%)	8.519	1.123–64.640	0.038
Creatinine, mg/dl	1.0 ± 0.2	1.0 ± 0.2	1.285	0.285–5.789	0.744
Glomerular filtration rate, ml/min	80.3 ± 16.8	74.5 ± 18.0	0.982	0.964–1.000	0.053
ERAT	11 (23.4%)	132 (82.5%)	15.429	7.010–33.955	< 0.001
Procedural data					
Procedure time, min	178.3 ± 46.5	181.9 ± 45.1	1.002	0.994–1.009	0.632
Radiofrequency time, min	64.5 ± 20.3	66.5 ± 18.1	1.006	0.988–1.024	0.506
Pulmonary vein isolation, *n* (%)	47 (100%)	160 (100%)	–	–	> 0.999
Substrate ablation, *n* (%)	46 (97.9%)	154 (96.3%)	0.558	0.065–4.754	0.594
Linear lesions, *n* (%)	9 (19.1%)	17 (10.6%)	0.502	0.207–1.214	0.126
CL difference between baseline and after substrate ablation, ms	33.4 ± 22.3	35.5 ± 27.6	1.003	0.988–1.019	0.681
Termination to AT or SR, *n* (%)	16 (34%)	26 (16.2%)	0.376	0.180–0.784	0.009
Termination to AT, *n* (%)	8 (17%)	20 (12.5%)	0.696	0.285–1.702	0.427
Termination to SR, *n* (%)	15 (31.9%)	15 (9.3%)	0.221	0.098–0.497	< 0.001

Multivariate analysis for late recurrence			
Predictor	Odds ratio	95% Confidence interval	*P* value
Age	1.042	0.976–1.113	0.220
Male	0.794	0.234–2.694	0.711
ERAT	16.828	6.184–45.797	< 0.001
LA area	1.020	0.936–1.112	0.647
AF history	1.006	0.995–1.018	0.286
AF duration	1.028	0.977–1.083	0.285
Mitral valve insufficiency, moderate or severe	6.390	0.659–61.996	0.110
Hypertension	1.086	0.284–4.148	0.904
Diabetes mellitus	3.183	0.576–17.574	0.184
Implanted device	6.310	0.386–103.253	0.184
CHA_2_DS_2_VASc Score	1.119	0.612–2.046	0.715
Glomerular filtration rate	1.007	0.975–1.040	0.672
Termination to SR	0.052	0.003–0.851	0.038
Termination to AT or SR	5.313	0.418–67.540	0.198

*ACEi* angiotensin-converting-enzyme inhibitors; *ARB* angiotensin II receptor type 1 blockers; *AT* atrial tachycardia; *BMI* body mass index; *CL* cycle length; *ERAT* early recurrence of atrial tachyarrhythmia; *LV-EF* left-ventricular ejection fraction; *SR* sinus rhythm; *TIA* transient ischemic attack

*Non-normally distributed continuous variables are expressed as median and interquartile range (25th and 75th percentile)

ERAT was observed at a significantly higher rate in patients with LR (82.5 vs. 23.4%, *P* < 0.001) than in patients without LR. The rate of intraprocedural termination to SR was significantly lower in the LR group (9.3 vs. 31.9%, *P* < 0.001).

On multivariate analysis, ERAT (OR 16.8, 95% CI 6.184–45.797, *P* < 0.001) was the only independent positive predictor of LR. Intraprocedural termination to SR (OR 0.052, 95% CI 0.003–0.851, *P* = 0.038) was identified as the only independent negative predictor of LR.

### Association of ERAT with long-term outcomes

**Fig. 2:**
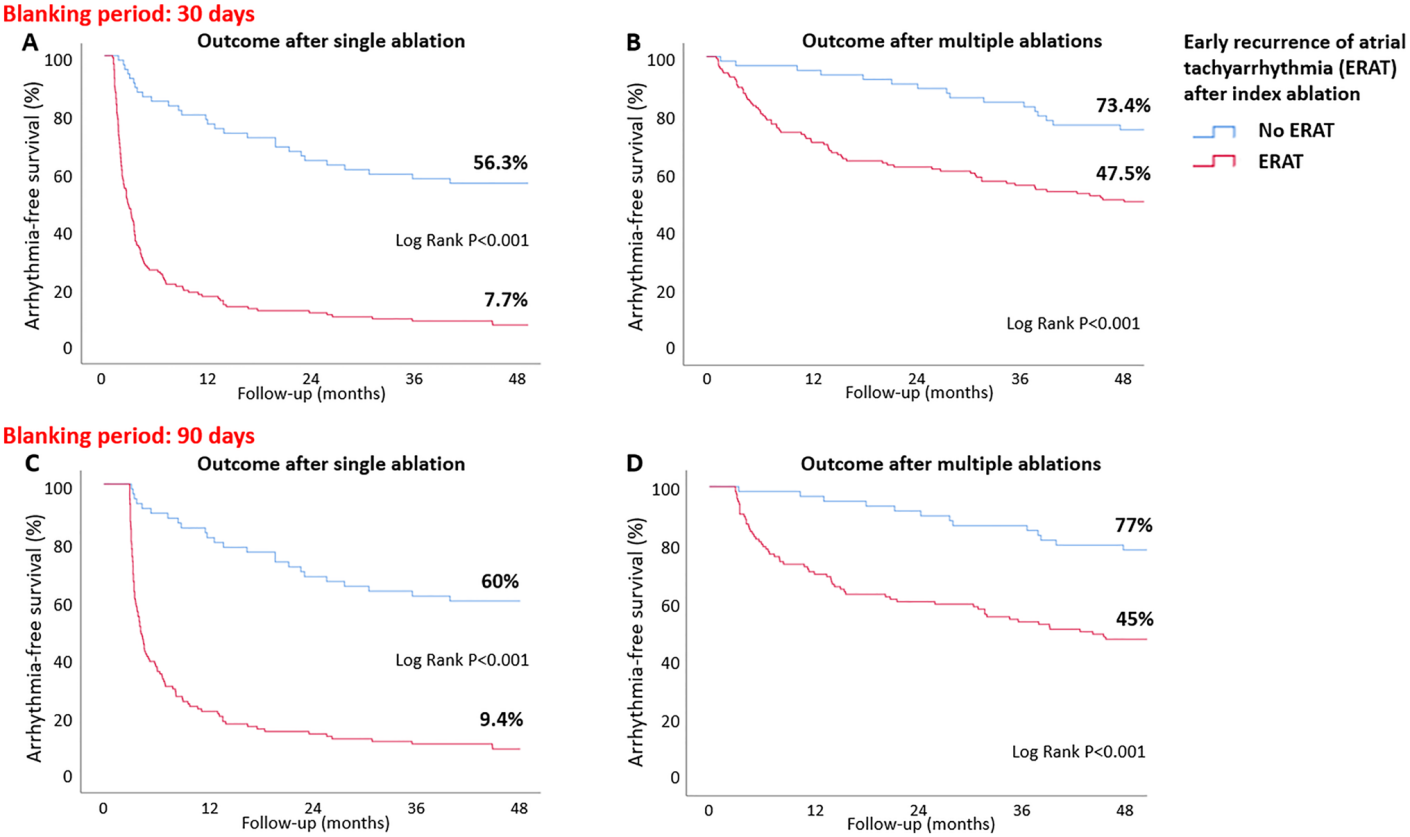
Freedom from any atrial arrhythmia off AAD (**A**) after a single ablation procedure and (**B**) after multiple ablation procedures (1.5 ± 0.8 vs. 2.3 ± 1.0, *P* < 0.001) in patients without and with ERAT (using a blanking period of 30 days; *n* = 207). Freedom from any atrial arrhythmia off AAD (**C**) after a single ablation procedure and (**D**) after multiple ablation procedures (1.5 ± 0.8 vs. 2.1 ± 1.0, *P* < 0.001) in patients without and with ERAT (using a blanking period of 90 days; subgroup analysis, *n* = 177)

**Fig. 3:**
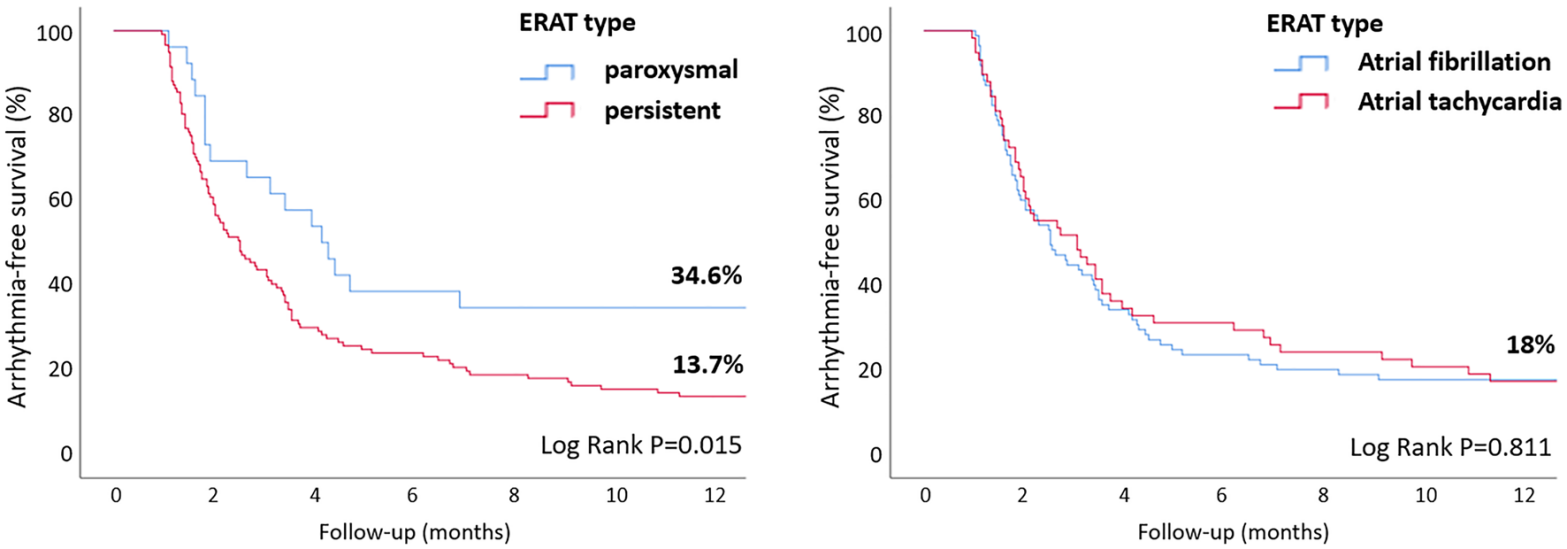
Freedom from any atrial arrhythmia off AAD after a single ablation procedure in patients with paroxysmal (26/143) or persistent (117/143) ERAT (**A**). Freedom from any atrial arrhythmia off AAD after a single ablation procedure in patients with atrial fibrillation (86/143) or atrial tachycardia (57/143) as ERAT (**B**)

Arrhythmia-free survival in patients with and without ERAT was assessed by Kaplan–Meier analysis (Fig. 2A). Freedom from any atrial arrhythmia off AAD 12 months after single ablation procedure was significantly higher in patients without vs. with ERAT (76.6 vs. 17.5%, *P* < 0.001) and in patients with paroxysmal vs. persistent ERAT (34.6 vs. 13.7%, *P* = 0.015) (Fig. 3). ERAT type (AF vs. AT) did not have an impact on single-procedural outcome.

Freedom from ERAT after the index procedure was associated with higher multi-procedural success rates (95.3 vs. 70.6% at 12 months, *P* < 0.001), as shown in Fig. 2B. There was no correlation between the timing of first ERAT manifestation and LR rates (Table 3).

**Table 3:** Late recurrence according to first ERAT occurrence

ERAT Timing	LR, %	Hazard ratio*	95% Confidence interval*	*P* value
No ERAT (*n* = 64)	43.8	N/A	N/A	N/A
ERAT during first week (*n* = 83)	95.2	6.9	4.39–10.87	< 0.001
ERAT during second week (*n* = 16)	87.5	3.6	1.91–6.95	< 0.001
ERAT during third week (*n* = 12)	83.3	3.6	1.73–7.40	0.001
ERAT during fourth week (*n* = 7)	100.0	5.6	2.41–12.82	< 0.001

*n* = 182. Exact ERAT timing could be determined in 118/143 (82.5%) patients. LR rate (%) was not statistically different when compared between the four ERAT groups (week 1, 2, 3 and 4; *P* = 0.296). In a separate Cox regression analysis, ERAT within 48 h was associated with a HR of 7.1 (CI 4.394–11.405; *P* < 0.001) and ERAT occurring later than 48 h with a HR of 4.7 (CI 2.92–7.409; *P* < 0.001)

*ERAT* early recurrence of atrial tachyarrhythmia, *LR* late recurrence

*No ERAT is the reference category

A total of 101/117 (86%) patients with persistent ERAT received early external cardioversion during the blanking period. Overall recurrence rate after external cardioversion was 83% within the first year and 94% during the entire follow-up period. Recurrence after external cardioversion occurred after a median of 35 days (IQR 9; 85).

### Late recurrence after a blanking period of 90 days: a subgroup analysis

In a subgroup analysis (*n* = 177), LR rate was reassessed by assuming a blanking period of 90 days instead of 30 days. A total of 30/207 patients (15%) having received a redo procedure before day 90 due to uncontrolled atrial arrhythmia were excluded from this analysis.

ERAT occurred in 117/177 (66.1%) patients during the first 90 days with 114/117 (97.4%) cases being first observed within the first 30 days. Among the three ERAT cases that were identified additionally (ERAT timing on day 66, 71 and 81), the LR rate was 100%. Single- and multi-procedural freedom from any atrial arrhythmia off AAD is shown as Kaplan–Meier analyses in Fig. 2C and D.

## Discussion

### Main findings

This is to the best of our knowledge the first study analyzing ERAT and its association with single- and multi-procedural long-term outcomes in a large, purely persistent AF patient cohort off AAD therapy.

Patients experiencing ERAT within 30 days after ablation showed a significantly higher risk of LR compared to patients without ERAT**,** with nearly all ERAT patients (> 90%) also presenting LR. ERAT was a strong independent predictor for LR. Intraprocedural termination to SR independently predicted freedom of any arrhythmia. The absence of ERAT after the index ablation was associated with superior multi-procedural outcomes.

### Incidence and characteristics of ERAT

ERAT is a common phenomenon after CA for AF with a reported incidence of 16–65% and a pooled estimate of 37.8% [1]. The significance of ERAT in patients with persistent AF is not well characterized, since prognostic value and characteristics of early recurrences are predominantly based on data from patients with paroxysmal AF [1–3, 14, 15]. A recent meta-analysis investigating the predictive value of ERAT after RFCA included data which originates from ~ 90% paroxysmal AF patients, while the persistent AF population remains clearly underrepresented [9]. In the paroxysmal AF cohort, there is increasing evidence that ERAT after PVI predicts LR [2, 8, 16].

The present study found an ERAT incidence of 69.1% during the first month following CA for persistent AF. ERAT rates are higher in persistent compared to paroxysmal AF [10–12]. The relatively high ERAT rate in our study may be explained by the fact that all patients remained off AADs, whereas in previous studies AADs were commonly restarted post-ablation. It has been reported that transient AAD use during the blanking period significantly reduces ERAT without preventing LR at 6 and 12 months [17, 18]. Since AADs rather seem to “delay” atrial arrhythmia relapses, current data more likely reflect the natural course of ERAT occurrence during the blanking period. Furthermore, routine use of AADs post-ablation is not generally recommended by current guidelines, since its benefit remains unclear [7]. Substrate ablation in addition to PVI may have also impacted ERAT rates in the current study. However, it was previously reported that additional substrate ablation does not increase ERAT rates [19].

In accordance with previous studies [10, 11, 15], ERAT showed the highest incidence in the first week post-ablation, occurring in nearly half of the patients within the first 48 h. Early recurrences were predominantly persistent, while spontaneous conversion to SR was observed in only 18% of patients. This is in strong contrast to previous reports from paroxysmal AF cohorts, where the prevalence of paroxysmal ERAT gradually diminished over the weeks following ablation [10, 15].

We also found a different correlation between ERAT timing and LR than previously described for paroxysmal AF, where the risk of relapse is known to gradually increase with later ERAT occurrence [2, 8, 10]. In our cohort, the risk of LR was rather equally high regardless of ERAT onset. Remarkably, the overwhelming majority of patients (95.2%) experiencing ERAT within the first week went on to have late arrhythmia recurrences. This data suggests a rather dichotomous relationship between ERAT and the risk of LR in patients with persistent AF.

Early cardioversion is thought to promote reverse remodeling processes in patients with ERAT [20]. In our study, 86% of patients with persistent ERAT received cardioversion during the blanking period. Despite restoration of SR by early cardioversion, there was a high rate of LR (83% within 1 year and 94% by the end of follow-up), which is in line with previous reports [20].

In summary, the vast majority of persistent AF patients with ERAT did not experience spontaneous conversion to SR during the blanking period and presented LR despite early cardioversion. The type of LR (62.5% AF and 37.5% AT) mainly corresponded to ERAT type, as reported previously [11], which suggests that ERAT is not a result of solely transient mechanisms.

### Predictive value of ERAT

Patients with ERAT showed a significantly higher rate of LR compared to patients without ERAT (92.3 vs. 43.8%, *P* < 0.001). On multivariate analysis, ERAT was the only independent positive predictor of LR (OR 16.8, *P* < 0.001). This adds to the growing body of evidence that ERAT is highly predictive of LR in paroxysmal AF [2, 8]. Rather surprisingly, the absence of ERAT following index ablation was also associated with superior multi-procedural outcomes in this study; patients without ERAT were to a significantly higher rate arrhythmia-free off AADs after less ablation procedures.

ERAT has been generally considered to be of transient nature due to reversible mechanisms involving an inflammatory response [4], autonomous imbalance [5] and time needed for lesion maturation post-ablation [6]. However, recent studies suggest that inflammation is likely limited to the first month post-ablation [21] and that ERAT is in fact significantly associated with PV reconnection [3, 14], hence challenging the concept that early relapses are of transient nature. Indeed, the traditional 3-month blanking period has been increasingly called into question by several reports which proposed limiting the blanking period to 2 months [10], 50 days [2], 46 days [12] and 23 days [11]. Notably, in the present study, nearly all (97.4%) ERAT episodes initially manifested in the first 30 days post-ablation.

In line with these findings, our subgroup analysis showed that extending the blanking period from 30 to 90 days did not impact LR rates. Therefore, “delayed cure” in months 2 and 3 after PVI may not be a realistic expectation in the persistent AF patient cohort. Thus, it may seem reasonable to schedule re-ablation in patients with ERAT.

Patients with persistent AF and ERAT are at very high risk of developing LR irrespective of ERAT onset. We, therefore, speculate that different pathomechanisms are causal for ERAT in paroxysmal and persistent AF patients. While ERAT in paroxysmal AF may be driven by transient mechanisms within the first weeks, ERAT in persistent AF could predominantly be caused by atrial substrate (involving atrial enlargement, scarring and slow conduction zones) which continues to be a driver of AF despite first ablation. This hypothesis is supported by the time-dependency between ERAT and LR in paroxysmal AF [2, 8, 10] as opposed to the rather dichotomous relationship we found in persistent AF patients. The association of ERAT with inferior multi-procedural outcomes is also suggestive for a non-transient cause of early recurrences.

Conversely, patients without ERAT had significantly smaller LA areas and thus likely present a lower degree of atrial electroanatomic remodeling and hemodynamic burden [2]. In addition, LA area was the only independent predictor of ERAT in the current study.

The maintenance of SR after ablation may prevent functional and structural remodeling and thus improve outcomes [7]. Patients with paroxysmal ERAT presented lower LR rates than those with persistent ERAT and intraprocedural termination to SR negatively predicted LR (OR 0.052, P = 0.038), as previously reported [13]. This suggests that modification of critical atrial substrate (PV foci, slow conduction areas) during ablation may play a key role in improving procedural outcomes.

### Outlook and clinical implications

The optimal management strategy for ERAT remains challenging [7]. Based on current results, ERAT in persistent AF should be distinguished from ERAT in paroxysmal AF, as it shows different characteristics and prognostic significance. We propose that patients experiencing ERAT during a 30-day blanking period should be informed of being at high risk of developing LR, considering the overwhelming LR rate of > 90% in this population. Early re-ablations were previously shown to significantly reduce LR rates [16, 22], however, safety and appropriate timing of repeat procedures remain to be addressed in future prospective studies.

### Limitations

This study is a retrospective single-center analysis with the inherent limitations of this design. ERAT was determined using a clinically feasible approach. However, implantable event recorder devices or transtelephonic monitoring could have provided a more accurate ERAT detection. Most patients received substrate ablation in addition to PVI; therefore, results may not be directly applicable to patients receiving PVI alone. However, we found no association between RF time or additional substrate ablation and ERAT occurrence. Furthermore, substrate ablation in addition to PVI was previously shown not to increase ERAT rate [19].

### Conclusions

ERAT following first catheter ablation (PVI ± atrial substrate ablation) for persistent AF is a strong independent predictor of late arrhythmia recurrence, which challenges the assumption that ERAT represents merely a transient phenomenon. A limited blanking period of 30 days may be sufficient to determine long-term rhythm outcomes after persistent AF ablation.

## References

[1] Andrade JG, Khairy P, Verma A, Guerra PG, Dubuc M, Rivard L (2012). Early recurrence of atrial tachyarrhythmias following radiofrequency catheter ablation of atrial fibrillation. Pacing Clin Electrophysiol.

[2] Willems S, Khairy P, Andrade JG, Hoffmann BA, Levesque S, Verma A (2016). Redefining the blanking period after catheter ablation for paroxysmal atrial fibrillation: insights from the advice (adenosine following pulmonary vein isolation to target dormant conduction elimination) trial. Circ Arrhythm Electrophysiol.

[3] Mujović N, Marinković M, Marković N, Vučićević V, Lip GYH, Bunch TJ (2018). The relationship of early recurrence of atrial fibrillation and the 3-month integrity of the ablation lesion set. Sci Rep.

[4] Grubman E, Pavri BB, Lyle S, Reynolds C, Denofrio D, Kocovic DZ (1999). Histopathologic effects of radiofrequency catheter ablation in previously infarcted human myocardium. J Cardiovasc Electrophysiol.

[5] Hsieh MH, Chiou CW, Wen ZC, Wu CH, Tai CT, Tsai CF (1999). Alterations of heart rate variability after radiofrequency catheter ablation of focal atrial fibrillation originating from pulmonary veins. Circulation.

[6] Fenelon G, Brugada P (1996). Delayed effects of radiofrequency energy: mechanisms and clinical implications. Pacing Clin Electrophysiol.

[7] Calkins H, Hindricks G, Cappato R, Kim Y-H, Saad EB, Aguinaga L (2017). 2017 HRS/EHRA/ECAS/APHRS/SOLAECE expert consensus statement on catheter and surgical ablation of atrial fibrillation. Europace.

[8] Kalinsek TP, Kottmaier M, Telishevska M, Berger F, Semmler V, Popa M (2020). Early recurrence after pulmonary vein isolation is associated with inferior long-term outcomes: insights from a retrospective cohort study. Pacing Clin Electrophysiol.

[9] Calkins H, Gache L, Frame D, Boo LM, Ghaly N, Schilling R, Deering T, Duytschaever M, Packer DL (2020). Predictive value of atrial fibrillation during the postradiofrequency ablation blanking period. Heart Rhythm.

[10] Themistoclakis S, Schweikert RA, Saliba WI, Bonso A, Rossillo A, Bader G (2008). Clinical predictors and relationship between early and late atrial tachyarrhythmias after pulmonary vein antrum isolation. Heart Rhythm.

[11] Alipour P, Azizi Z, Pirbaglou M, Ritvo P, Pantano A, Verma A (2017). Defining blanking period post-pulmonary vein antrum isolation. JACC Clin Electrophysiol.

[12] von Olshausen G, Uijl A, Jensen-Urstad M, Schwieler J, Drca N, Bastani H (2020). Early recurrences of atrial tachyarrhythmias post pulmonary vein isolation. J Cardiovasc Electrophysiol.

[13] Ammar S, Hessling G, Reents T, Paulik M, Fichtner S, Schön P (2013). Importance of sinus rhythm as endpoint of persistent atrial fibrillation ablation. J Cardiovasc Electrophysiol.

[14] Das M, Wynn GJ, Morgan M, Lodge B, Waktare JE, Todd DM (2015). Recurrence of atrial tachyarrhythmia during the second month of the blanking period is associated with more extensive pulmonary vein reconnection at repeat electrophysiology study. Circ Arrhythm Electrophysiol.

[15] Joshi S, Choi AD, Kamath GS, Raiszadeh F, Marrero D, Badheka A (2009). Prevalence, predictors, and prognosis of atrial fibrillation early after pulmonary vein isolation: findings from 3 months of continuous automatic ECG loop recordings. J Cardiovasc Electrophysiol.

[16] Andrade JG, Khairy P, Macle L, Packer DL, Lehmann JW, Holcomb RG (2014). Incidence and significance of early recurrences of atrial fibrillation after cryoballoon ablation: insights from the multicenter sustained treatment of paroxysmal atrial fibrillation (STOP AF) trial. Circ Arrhythm Electrophysiol.

[17] Darkner S, Chen X, Hansen J, Pehrson S, Johannessen A, Nielsen JB (2014). Recurrence of arrhythmia following short-term oral AMIOdarone after CATheter ablation for atrial fibrillation: a double-blind, randomized, placebo-controlled study (AMIO-CAT trial). Eur Heart J.

[18] Kaitani K, Inoue K, Kobori A, Nakazawa Y, Ozawa T, Kurotobi T (2016). Efficacy of antiarrhythmic drugs short-term use after catheter ablation for atrial fibrillation (EAST-AF) trial. Eur Heart J.

[19] Andrade JG, Macle L, Khairy P, Khaykin Y, Mantovan R, Martino GD (2012). Incidence and significance of early recurrences associated with different ablation strategies for AF: A STAR-AF substudy. J Cardiovasc Electrophysiol.

[20] Baman TS, Gupta SK, Billakanty SR, Ilg KJ, Good E, Crawford T (2009). Time to cardioversion of recurrent atrial arrhythmias after catheter ablation of atrial fibrillation and long-term clinical outcome. J Cardiovasc Electrophysiol.

[21] Lim HS, Schultz C, Dang J, Alasady M, Lau DH, Brooks AG (2014). Time course of inflammation, myocardial injury, and prothrombotic response after radiofrequency catheter ablation for atrial fibrillation. Circ Arrhythm Electrophysiol.

[22] Lellouche N, Jaïs P, Nault I, Wright M, Bevilacqua M, Knecht S (2008). Early recurrences after atrial fibrillation ablation: prognostic value and effect of early reablation. J Cardiovasc Electrophysiol.

